# Assessing the Impact of Climate Change on the Distribution of Lime (16srii-B) and Alfalfa (16srii-D) Phytoplasma Disease Using MaxEnt

**DOI:** 10.3390/plants10030460

**Published:** 2021-02-28

**Authors:** Amna M. Al Ruheili, Alaba Boluwade, Ali M. Al Subhi

**Affiliations:** 1 Department of Plant Science, College of Agriculture and Marine Science, Sultan Qaboos University, Muscat 123, Oman; alsubhia@squ.edu.om; 2 Department of Soil, Water and Agricultural Engineering, College of Agriculture and Marine Science, Sultan Qaboos University, Muscat 123, Oman; alaba@squ.edu.om

**Keywords:** future projection, distribution model, bioclimatic variables, witches’ broom disease

## Abstract

Witches’ broom disease has led to major losses in lime and alfalfa production in Oman. This paper identifies bioclimatic variables that contribute to the prediction of distribution of witches’ broom disease in current and future climatic scenarios. It also explores the expansion, reduction, or shift in the climatic niche of the distribution of the disease across the different geographical areas of the entire country (309,501 km²). The maximum entropy model (MaxEnt) and geographical information system were used to investigate the potential suitability of habitats for the phytoplasma disease. This study used current (1970–2000) and future projected climatic scenarios (2021–2040, 2041–2060, 2061–2080, and 2081–2100) to model the distribution of phytoplasma for lime trees and alfalfa in Oman. Bioclimatic variables were downloaded from WorldClim with ± 60 occurrence points for lime trees and alfalfa. The area under the curve (AUC) was used to evaluate the model’s performance. Quantitatively, the results showed that the mean of the AUC values for lime (16SrII-B) and alfalfa (16SrII-D) future distribution for the periods of 2021–2040, 2041–2060, 2061–2080, and 2081–2100 were rated as “excellent”, with the values for the specified time periods being 0.859, 0.900, 0.931, and 0.913 for 16SrII-B; and 0.826, 0.837, 08.58, and 0.894 for 16SrII-D respectively. In addition, this study identified the hotspots and proportions of the areas that are vulnerable under the projected climate-change scenarios. The area of current (2021–2040) highly suitable distribution within the entire country for 16SrII-D was 19474.2 km^2^ (7.1%), while for 16SrII-B, an area of 8835 km^2^ (3.2%) was also highly suitable for the disease distribution. The proportions of these suitable areas are very significant from the available arable land standpoint. Therefore, the results from this study will be of immense benefit and will also bring significant contributions in mapping the areas of witches’ broom diseases in Oman. The results will equally aid the development of new strategies and the formulation of agricultural policies and practices in controlling the spread of the disease across Oman.

## 1. Introduction

Citrus fruits are considered to be a major crop across the world, with about 60 million megatons each year produced according to the Food and Agriculture Organization of the United Nations (FAO) (2015) data. Witches’ broom diseases (WBDs) have negatively impacted several high-value agricultural products in Oman, such as acid lime (*Citrus aurantifolia* L.) trees and the alfalfa (*Medicago sativa L*) crop. These diseases are caused by phytoplasmas related to the 16SrII-B and 16SrII-D subgroups [1,2]. The 16SrII-D phytoplasma is more aggressive than the 16SrII-B type. Plants infected with 16SrII-D phytoplasma show many symptoms including phyllody and witches’ broom. Also, 16SrII-D phytoplasma infects wild plant hosts from different species. In Oman, 16SrII-D phytoplasma was reported in more than 25 plant hosts from economic crops and wild plants [3].

Phytoplasma are a group of bacteria that belongs to the Mollicutes class [4]. In Oman, leafhoppers—*Hishimonus phycitis*, *Austroagallia avicula*, and *Empoasca sp*.—and also *Diaphorina citri,* the Asian citrus psyllid, are the main and putative vectors in transmitting 16SrII-B [5] and 16SrII-D [6] phytoplasmas. The insect vectors of phytoplasmas were recorded from all areas infected with phytoplasmas in Oman [5,6]. The most common symptoms caused by phytoplasmas are witches’ broom, yellowing leaves, inhibited growth, big buds, leaf deformation, virescence, phyllody, purple color, bolting, the formation of bunchy fibrous secondary roots and discoloration, reducing yield, decline, and dieback [4,7]. A diseased tree takes about six months for its symptoms to develop [8].

Witches’ broom disease of acid lime trees (WBDL) is due to a phytoplasma infection that belongs to the 16SrII-B subgroup phytoplasma type, which was first found in Omani lime trees in the late 1970s and early 1980s [9]. The 16SrII-B subgroup phytoplasma, which is the causal agent of WBD, spread to other countries such as the United Arab Emirates (UAE) by 1989 and the southeastern part of Iran by 1997 [10]. As the disease progressed, the lime industry in Oman was severely impacted, and many trees were brought down to prevent the disease from spreading [11]. Acid lime trees constitute 4% of the fruit crops grown in Oman [12]. According to Al-Yahyai et al. [13], however, the spread of the disease is particularly acute in Oman, as 98% of acid limes were found to be infected with the 16SrII-B subgroup phytoplasma. In addition, more than half a million acid lime trees have been destroyed because of WBDL in Oman since 1990. This has resulted in the loss of more than 75% of acid lime production [14]. WBD affecting acid lime trees in Oman has, therefore, resulted in the loss of more than 50% of the cultivated acid lime area during the last four decades. WBDL (16SrII-B) kills acid lime trees in less than five years, and these trees’ production cannot be quickly restored [8].

Alfalfa is one of the main forage crops in Oman, with a value of US$120 million per year [6]. Alfalfa, which is produced in a limited capacity in Oman, is a reported host for the 16SrII-D phytoplasma disease. According to Khan et al. [15], phytoplasma disease results in a 25% loss in alfalfa production, leading to a loss of US$30 million per year. The main symptoms of infected alfalfa are an excessive increase in the number of shoots and the yellowing of leaves, which reduce the marketability of the crop [6]. 

Alfalfa witches’ broom (AlfWB), which affects crops across the world, was first reported in the 1990s from all regions of Oman [15]. Several causal agents have been documented for WBD in alfalfa, such as ‘*Candidatus Phytoplasma asteris*’ in the USA, ‘*Ca. Phytoplasma trifolii*’ in Canada, ‘*Ca. phytoplasma fraxini*’ in Argentina, and the 16SrII-D subgroup in Oman and Iran [3,16]. 16SrII-D phytoplasma causes WBD in Alfalfa in Saudi Arabia and the UAE [10,15]. In Oman, the WBD has been found all across the country, though fewer infections are reported from the southern part of Oman [11]. Due to the importance of acid lime trees and alfalfa crops to the Omani economy, this research focuses on exploring the environmental factors that contribute to phytoplasmas. Although disease identification, symptoms, and hosts have already been extensively researched, very few studies have been carried out to assess the results of climate change using modeling tools to create different climate-change scenarios and consider the host plants. Several anthropogenic practices contribute to producing greenhouse gases, which have changed the patterns of temperature and precipitation around the world [17]. Therefore, the primary aim of this study is focused on implementing MaxEnt to explore the environmental factors that contribute to the phytoplasmas that affect acid lime trees and alfalfa crops. The MaxEnt model is a robust and maximum entropy-based model that is capable of simulating the species’ spatial distribution using relevant environmental variables and species occurrence information.

## 2. Materials and Methods

### 2.1. Species Occurrence Data

**Figure 1:**
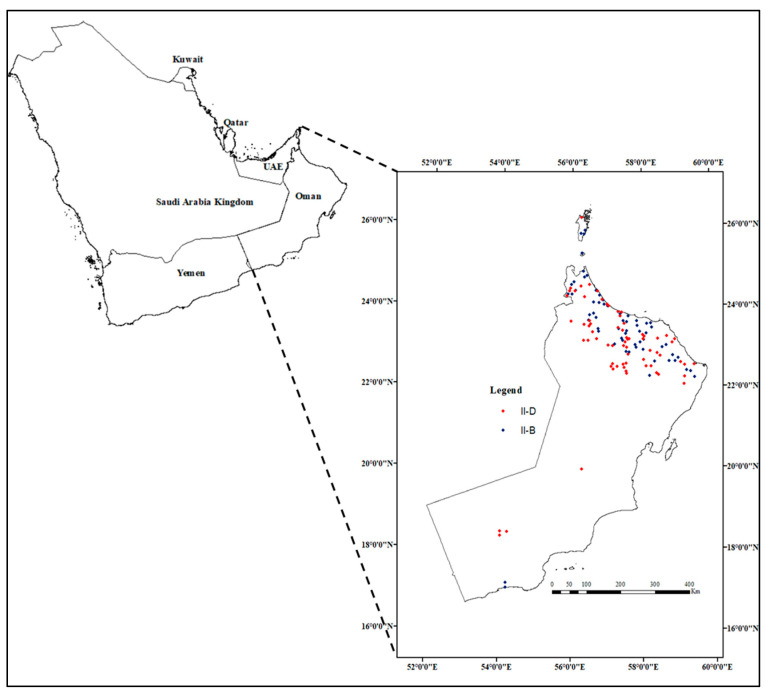
Occurrence data distribution for 16SrII-B (lime, blue color) and 16SrII-D (alfalfa, red color).

A total of 174 phytoplasma-symptomatic (pathogen incidence) samples of both acid lime trees and alfalfa plants were collected at various locations in North and South Oman during a survey conducted from 2015 to 2020. The identifications and classifications of phytoplasmas of acid lime trees and alfalfa infected with phytoplasma disease were proven using molecular techniques which were conducted to amplify the 16Sr RNA gene sequence of phytoplasmas in previous studies [3,18,19]. Polymerase chain reactions (PCR) of the 16Sr RNA gene were performed by using the forward primer P1 [20] and the reverse primer P7 [21] as direct PCR. The R16F2n/R16R2 primer pair was used for the nested PCR. The 16Sr DNA sequences results of infected acid lime trees and alfalfa plants samples showed that the phytoplasmas belong to 16SrII-B and 16SrII-D subgroups [1,3,18,19]. The data were gathered from various farms of acid lime trees infected with 16SrII-B phytoplasma (87 locations) and alfalfa infected with 16SrII-D phytoplasma (87 locations) (see Figure 1). The records of both the 16SrII-B and 16SrII-D subgroup phytoplasmas were cleaned by removing the point duplications, evaluating the coordination of the points, and removing false locations outside the geographic areas being studied. Then, spatial autocorrelation was conducted using the Species Distribution Model SDM toolbox in ArcMap 10.8 to achieve spatial independence of the data. The occurrence data were kept 10 km apart from each other to keep the maximum number of records [22].

### 2.2. Environmental Data

Twenty variables were taken into consideration for the purpose of this research. Of them, eleven were derived from the monthly temperature (bio1–bio11) and eight were from the monthly precipitation (bio12–bio19) in addition to the topographic elevation. The bioclimatic variables were acquired from the WorldClim dataset [23] at a 2.5 min resolution (about 5 km).

The bioclimatic variables obtained from WorldClim version 2.1 covering the period from 2020 to 2040 were downscaled using the CMIP6 model. The climatic data include nine global climate models (GCMs) and four shared socio-economic pathways (SSPs) for four periods: 2021–2040, 2041–2060, 2061–2080, and 2081–2100 and are available as a Geotif [24]. This study used SSP585 to extract the 19 bioclimatic variables (in Geotif data format) using R-Studio, and the average of the bioclimatic variables were used as an input for MaxEnt. The bioclimatic variables derived from WorldClim have been utilized by many researchers in predicting potential species distributions as influenced by changes in temperature and precipitation [25].

To avoid multicollinearity, the cross-correlations were run on the SDMtoolbox in ArcMap10.8. Multicollinearity is a type of regression analysis used to explore the connections between dependent and independent variables [26]. This study used Pearson’s correlation coefficient, r =0.8, which was selected as a cut-off threshold to remove strongly correlated bioclimatic variables for the 16SrII-B and 16SrII-D subgroup phytoplasmas. The results showed that there was no multicollinearity between the variables for 16SrII-B, and 16SrII-D bio2 was removed as it showed multicollinearity. Therefore, 20 environmental variables were used to model both the 16SrII-B and 16SrII-D subgroup phytoplasmas that included the digital elevation model (DEM) as shown in Table 1 and Table 2. A bold font was used to highlight the variables that contributed to the disease distribution.

### 2.3. Predictive Modeling

MaxEnt (maximum entropy, version 3.3.3k downloaded from http://www.cs.princeton.edu (accessed on 12 December 2020)) was used in this research to analyze and map the current (1970–2000) and future (2021–2040, 2041–2060, 2061–2080, and 2081–2100) potential distribution of the 16SrII-D and B subgroup phytoplasmas across Oman. The MaxEnt model uses occurrence points to estimate occurrence probabilities based on species data and bioclimatic variables. MaxEnt algorithms are based on computing the occurrence probability derived from randomly produced background points and species presence records and then judging the maximum entropy distribution. MaxEnt can include both continuous and categorical variables and control overlaps through the use of regularization parameters [27]. The advantage of MaxEnt is its ability to work better with low occurrence data than other species distribution models [28].

To make sure the predicted results are accurate in terms of modeling, a sampling bias layer was created in SDMtoolbox (v2.7) using a kernel density tool to limit the background points of the occurrence data and to identify the preferred locations within the area being surveyed. To confine the geographical distribution of a habitat or a species, MaxEnt uses five various separate attributes: linear, product, quadratic, hinge, and threshold [29].

### 2.4. Model Development and Validation

SDMtoolbox (v2.7) was used to assess the environmental variables that contribute to the distribution of 16SrII-D and 16SrII-B and the impact of climate change on future dispersal. SDMtoolbox is a Python-based ArcGIS toolbox developed in Duke University, the City College of New York, and Southern Illinois University and is used to study the spatial potential of areas suitable for the ecology, evolution and genetics of species [30]. SDMtoolbox is a series of Python scripts that have been developed to automate the spatial analysis process in ArcMap and Python [31]. This tool is embedded in the MaxEnt model.

The model produced the area under the curve (AUC), which was used to calculate the goodness of fit of the model. The AUC is represented in values from 0 to 1. Five categories are used to judge the model’s performance: the model is failing (0.5–0.6), poor (0.6–0.7), fair (0.7–0.8), good (0.8–0.9), or excellent (0.9–1) [32]. The contributions of the bioclimatic variables to the 16SrII-D and B phytoplasmas distribution were measured with the help of a jackknife test.

In addition to the usage of AUC to evaluate SDM’s fit with true presence and absence data, the true skill statistic (TSS = sensitivity + specificity − 1) is used, and it creates values ranging between −1 and 1. The produced values above zero indicate that the model’s performance is good [28]. The robustness of the model using the independent presence and absence of the dataset has values of 0.89, 0.78, and 0.67 for sensitivity, specificity, and TSS respectively. 

A regularization multiplier (RM) was used to select the best model, as different combinations of RMs produce different results. MaxEnt uses an RM to select the features that contribute the most to the model to reduce model overlapping [33]. The RM values used in this study were 0.5, 1, 1.5, 2, 2.5, and 3. Linear [L], quadratic [Q], product [P], threshold [T], and hinge [H] were set in MaxEnt in addition to other RM combinations to obtain the best model for 16SrII-D and 16SrII-B subgroup phytoplasmas.

To calculate the connections between the predicted probabilities for 16SrII-D and 16SrII-B phytoplasmas and each of the environmental variables, the ‘response curves’ were used. With the help of a jackknife test, the relative influences of different environmental variables on the 16SrII-D and 16SrII-B phytoplasmas distributions were calculated. ‘Jackknife’ produces the percentage contribution to estimate the contribution of a particular variable to the model and the permutation’s importance to show the extent to which the model depends on that variable [27].

### 2.5. SrII-D and SrII-B Phytoplasmas Model Assessment

So far as the 16SrII-D subgroup phytoplasma is concerned, this study tested 40 combinations of RMs and feature types to select the best model for the distribution of the disease. All the tested models did very well in their projections—all of them had low omission rates at 10% and excellent AUC values. The lowest ORs at 10% were 0.11, whereas the highest AUC value was 0.874.

For the 16SrII-B subgroup phytoplasma, this study tested 59 combinations of RMs and feature types to select the best model for the distribution of the disease. Once again, all the tested models performed very well in their projections—they had low omission rates at 10% and excellent AUC values. The lowest ORs at 10% were 0.067, whereas the highest AUC value was 0.914 at a standard deviation of 0.015.

The best model for 16SrII-D phytoplasma includes seven environmental variables—linear [L] and quadratic [Q] features, and RM=3. In contrast, for 16SrII-B phytoplasma, it includes only six environmental variables—linear [L], quadratic [Q], and product [P] features, and RM =0.5. 

## 3. Results

### 3.1. Model Validation and Influencing Bioclimatic Variables

**Figure 2:**
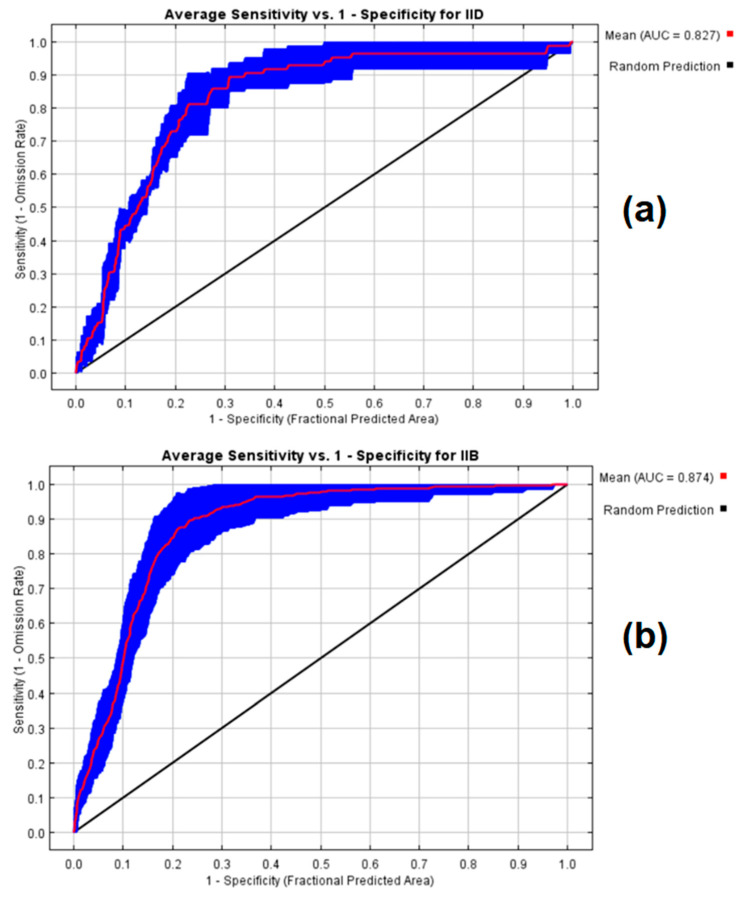
The ROC curve for II-D & II-B showing the AUC for (**a**) Alfalfa (16SrII-D) and (**b**) lime (16SrII-B). The envelope around the mean AUC (plus/minus one standard deviation) is shown in blue color. For 16SrII-D phytoplasma, the seven bioclimatic variables that were influential in the spatial distribution of phytoplasmas in Alfalfa were isothermality (bio3, contributed 29.7%), mean diurnal range (bio2, contributed 25.6%), seasonal precipitation (bio15, contributed 13.1%), mean temperature of the wettest quarter (bio8, contributed 10.1%), precipitation in the warmest quarter (bio18, contributed 7%), precipitation in the driest month (bio14, contributed 5.6%), and topography (DEM, contributed 5.6%).

For the period between 2021 and 2040, the results for the suitability and distribution of AlfWB phytoplasma disease (16SrII-D) of alfalfa were found to be highly significant, where the average AUC for the 25 replicate runs is 0.827. The highest value of the AUC for AlfWB (16SrII-D) disease is 0.874 for the training sample and 0.860 for the test sample at a standard deviation of 0.029. The average AUC for WBDL phytoplasma disease of acid lime (16SrII-B) was 0.874, and for the test sample, it was 0.910 at a standard deviation of 0.045 (Figure 2). This indicated that the bioclimatic variables set for 16SrII-D and 16SrII-B subgroup phytoplasmas, which were used to predict the model and interpret its potential suitability, worked well and were highly accurate.

In contrast, for the 16SrII-B variant, the five main bioclimatic variables that significantly contributed to the spatial distribution of phytoplasmas in lime trees were isothermality (bio3, contributed 36.9%), temperature annual range (bio7, contributed 24.1%), minimum temperature of the coldest month (bio6, contributed 9.8%), seasonal precipitation (bio15, contributed 7.9%), and precipitation of the driest month (bio14, contributed 5.9%). Meanwhile, the precipitation of the warmest quarter (bio18, contributed 3.1%) and the mean temperature of the driest quarter (bio9, contributed 3.2%) were less influential. 

The mean of the AUC values for the future distribution of 16SrII-B and 16SrII-D phytoplasma for the periods of 2021-2040, 2041–2060, 2061–2080, and 2081–2100 are shown in Table 3. The AUC values for 16SrII-B, which were ranked as “excellent”, were 0.859, 0.900, 0.931, 0.913 for 2021-2040, 2041–2060, 2061–2080, and 2081–2100 respectively. The corresponding values for 16SrII-D were 0.826, 0.837, 08.58, and 0.894 respectively. The results of the simulations, therefore, indicate the model’s reliability in analyzing the impact of climate change on the distribution of phytoplasma disease for the 16SrII-B (WBDL) and 16SrII-D (AlfWB) variants.

### 3.2. Current and Future Potential Suitable Habitats under Different Climatic Scenarios

**Figure 3:**
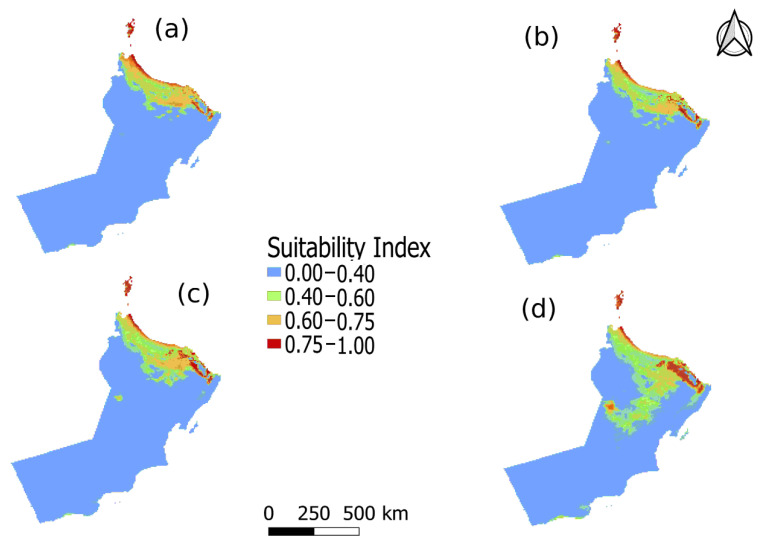
Potentially suitable climatic distribution of 16SrII-B phytoplasma under different climate-change scenarios in Oman for (**a**) 2021–2040, (**b**) 2041–2060, (**c**) 2061–2080, and (**d**) 2081–2100.

**Figure 4:**
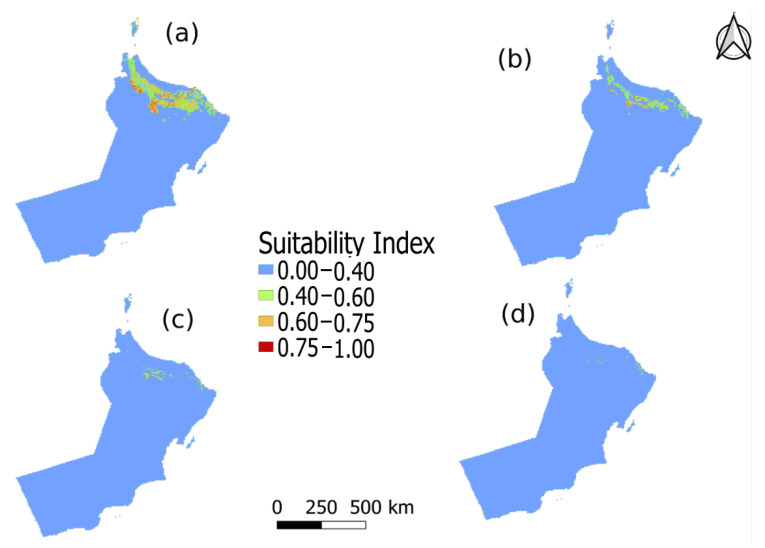
Potentially suitable climatic distribution of 16SrII-D phytoplasma under different climate-change scenarios in Oman for (**a**) 2021–2040, (**b**) 2041–2060, (**c**) 2061–2080, and (**d**) 2081–2100.

The predicted suitable future distributions for 16SrII-D and 16SrII-B subgroup phytoplasma, under current and projected climate-change scenarios, for the periods of 2021–2040, 2041–2060, 2061–2080, and 2081–2100 are shown in Figure 3 and Figure 4.

Figure 3 and Figure 4 shows the major regions that are most potentially suitable for the distribution of AlfWB (16SrII-D) and WBDL (16SrII-B) diseases. The total suitable habitat includes ill-suited habitat (0–0.40), poorly suited habitat (0.40–0.60), moderately suitable habitat (0.60–0.75), and highly appropriate habitat (0.75–1.0). The highly suitable habitats (0.75–1.0) for both 16SrII-B and 16SrII-D phytoplasmas were primarily located in the northern part of Oman. Under the current climatic conditions, 10,211.4 km^2^ (3.74%) of the area is highly suitable for the distribution of 16SrII-B phytoplasma, and most of the impacted area of 306,188.37 km^2^ (8.4%) is located along the coastal area, while the 16SrII-B phytoplasma disease affects more crops in the southern parts of the country in comparison to the 16SrII-D variant. However, 16SrII-D shows an increase at a moderately suitable habitat (0.60–0.75) during 2021–2040, with an extensive distribution across Oman to about 8.57% of the area. In the same period, 16SrII-B phytoplasma shows an increase in moderately suitable habitat to about 7.22% of the area along the coastal area at the northern part of Oman. The potential distribution of disease under future climatic scenarios during the periods of 2021-2040, 2041–2060, 2061–2080, and 2081–2100 are shown in Figure 3 and Figure 4.

**Figure 5:**
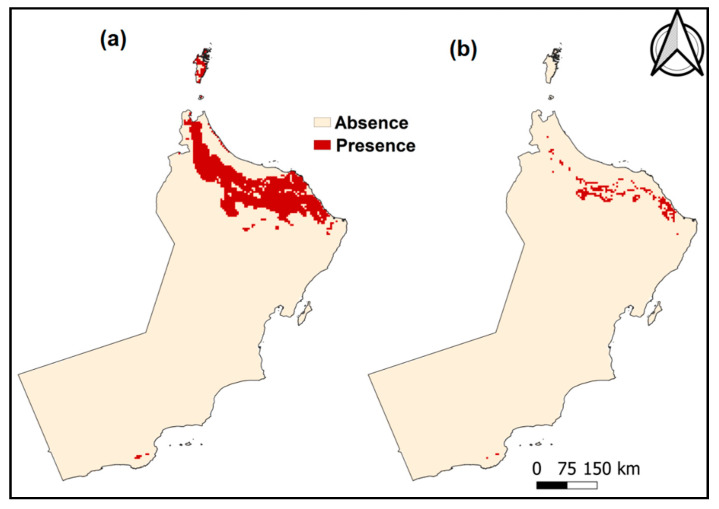
Presence and absence in the habitat for 16SrII-D using the maximum training sensitivity plus specificity threshold for (**a**) 2061–2080, (**b**) 2021–2040.

**Figure 6:**
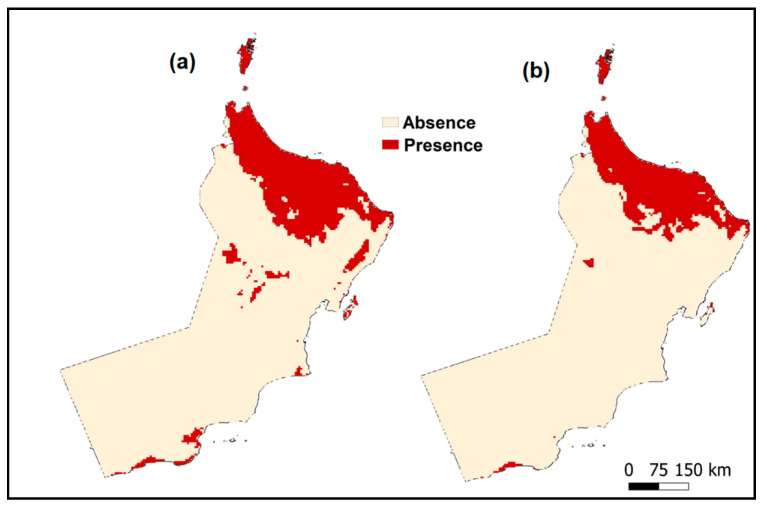
Presence and absence in the habitat of 16SrII-B using the maximum sensitivity plus specificity threshold for (**a**) 2061–2080, (**b**) 2021–2040.

Moreover, the output of the maximum training sensitivity plus specificity from MaxEnt used as a threshold to produce a binary map of the presence and absence of 16SrII-D and 16SrII-B as recommended by Hu and Jiang’s study [34]. Figure 5 and Figure 6 show the presence and absence in the habitat of 16SrII-D and 16SrII-B.

Table 4 shows the percentage of distributional changes between two binaries of current and future climate scenarios for 16SrII–B phytoplasma. The distribution change is categorized as gain, no change, and loss classes. The overall result showed an increase in all classes with a maximum increase in the gain class of 24.74% in the period of 2081–2100 and minimum loss of about 0.01% in the period of 2061–2080. On the other hand, Table 5 shows the percentage of distributional changes for 16SrII–D phytoplasma where a minor increase in the gain and the loss classes was observed with a maximum increase of 0.08% and a minimum loss of about 11.56% in the period of 2021–2040. That being said, the period of 2021–2040 showed a higher percentage of no change in the habitat distribution of the disease by 88.36% and a maximum loss of the disease habitat distribution by 21.51% in the period of 2081–2100.

Concerning 16SrII-D phytoplasma, compared to the current distribution, in future climatic scenarios during the periods of 2021–2040, 2041–2060, 2061–2080, and 2081–2100, the total area of moderately suitable habitat (0.60–0.75) for the distribution of disease would increase by 1.58%, decrease by −3.77%, decrease by −5.23%, and decrease by −6.42% respectively. The total area of highly suitable habitat (0.75–1.0) would increase by 1.38%, decrease by −2.89%, decrease by −4.92%, and decrease by −5.29% respectively (Table 6). Also, the areas of poorly suitable habitat (0.40–0.60) would first increase by 21.95% and then increase by 5.01%, increase by 1.2%, and decrease by −3.26% respectively (Table 6). Moreover, the total area of unsuitable habitat (0.0–0.40) for the distribution of phytoplasmas would decrease by −24.91%, and increase by 1.66%, 8.94%, and then by 14.96% respectively (Table 6).

On the other hand, for 16SrII-B phytoplasma, the results showed that in comparison to the current distribution, the total area of moderately suitable habitat (0.60–0.75) for the disease distribution would increase by 0.76% between 2021 and 2040, increase by 0.04% between 2041 and 2060, increase by 0.86% between 2061 and 2080, and increase by 1.25% from 2081 to 2100. During these three time periods, the total area of highly suitable habitat (0.75–1.0) would decrease by −0.53%, decrease by −1.32%, decrease by −0.67%, and increase by 0.09% respectively (Table 7). In addition, the area of poorly suitable habitat (0.40–0.60) would increase by 0.80, 2.59, 3.76, and 15.69% respectively (Table 7). Moreover, the total area of unsuitable habitat (0.0–0.40) for the distribution of phytoplasmas would decrease across the various climate projections by −1.03, −1.30, −3.95, and −17.03% respectively (Table 7).

**Figure 7:**
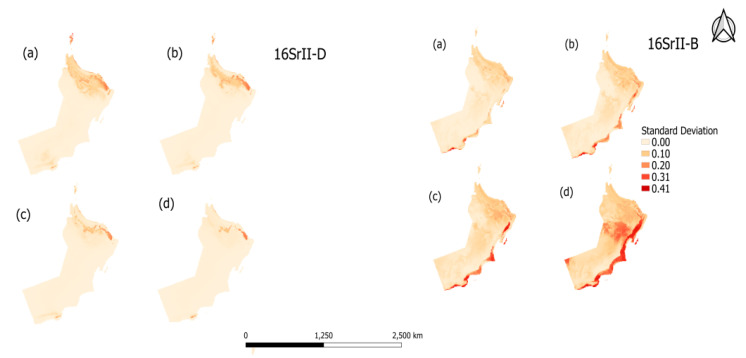
The standard deviation maps of 16SrII-D and 16SrII-B habitat distribution in maximum entropy (MaxEnt) for climatic projections (**a**) 2021–2040 (**b**) 2041–2060 (**c**) 2061–2080, and (**d**) 2081–2100.

Standard deviation (SD) was used to quantify the error associated with the climatic projections as shown in Figure 7. The climatic projection for both the 16SrII-B and 16SrII-D subgroup phytoplasma for the period 2061–2080 and 2081–2100 shows higher SD variability than the period 2021–2040 and 2041–2060 in habitat suitability. 

## 4. Discussion

This study was the first of its kind to look into the impacts created by bioclimatic factors in association with projected climate-change scenarios on the geographical distribution of phytoplasmas for 16SrII-D (AlfWB) and 16SrII-B (WBDL) diseases across Oman using MaxEnt modeling. MaxEnt modeling has been frequently used by many researchers due to its rapid processing ability and its capacity to provide comprehensive results concerning the current and future occurrences of a target species [35].

Al-Ghaithi et al. [8] documented the possibility of the impact of environmental factors on the distribution of 16SrII-B phytoplasmas—the causal agent of WBDL disease in acid lime trees—but they were unable to identify these environmental factors. This study modeled the potential distribution of 16SrII-D and 16SrII-B subgroup phytoplasma diseases and showed the possibly impacted area under both current and future climate-change scenarios. The MaxEnt model resulted in an “excellent” rating for the AUC value of 0.826 for AlfWB (16SrII-D) and 0.859 for WBDL (16SrII-B) under the 2021–2040 climate scenario. Donkersley et al. [36] suggested establishing nurseries in areas where most of the phytoplasma infection of acid lime trees can be found. 

Moreover, the MaxEnt model provided values for all the projected climatic scenarios and also predicted the potential distribution of the disease under different climatic conditions. For 16SrII-B phytoplasma disease, it provided AUC values of 0.8598, 0.900, 0.931, and 0.913 for the periods of 2021–2040, 2041–2060, 2061–2080, and 2081–2100 respectively. For the 16SrII-D phytoplasma disease, the MaxEnt model provided AUC values of 0.8268, 0.837, 0.858, and 0.894, respectively, for the same four periods. These results are available as a baseline for future studies that focus on mapping the distribution of other phytoplasma types of other hosts in Oman. Due to MaxEnt’s ability to detect localities with similar conditions for occurrence, this study provided a good proxy for a suitable habitat for the 16SrII-B and 16SrII-D vectors.

The simulation of the potential distribution of 16SrII-B and 16SrII-D phytoplasmas are based on data obtained from native regions rather than data from exotic areas. The simulation in this study should, therefore, be regarded as a realized niche instead of a fundamental niche [37]. It is worth mentioning, moreover, that this study is based on climatic variables (20 climatic variables: bio1–bio19 and DEM) rather than on other abiotic factors, such as soil, hydro-geology, and other variables. According to Li et al. [38], bioclimatic variables should be considered as critical factors in controlling the redevelopment and spread of natural populations. For example, a study conducted by Nagler et al. [39] showed the impact and the significant contribution of bioclimatic variables on the distribution of *Elaeagnus angustifolia*. 

The result of the MaxEnt modeling has revealed and predicted the different distribution of suitable habitats for 16SrII-B and 16SrII-D phytoplasmas of WBDL and AlfWB diseases. As shown on the map in Figure 3, the coastal area had the potential for the distribution of 16SrII-B phytoplasma across the various climatic scenarios, even in the southern part of the country. Although some of the collected occurrence samples were asymptotic, the area along the coast will still be a hotspot for the disease. However, for 16SrII-D phytoplasma, it was found that while the coastal area in the north was a highly suitable habitat for the distribution of the disease, the southern coast of Oman was not so. Nevertheless, the areas of moderately suitable and highly suitable habitat kept decreasing, and their distribution reduced across Oman for the period for all future scenarios except for the period between 2021 and 2040. Global warming will, therefore, greatly influence the distribution of 16SrII-D phytoplasma disease by causing shifts or contractions in the ranges of the disease in specific areas (Figure 4). On the other hand, MaxEnt predictions showed that potentially highly suitable climatic distributions for 16SrII-B phytoplasmas will expand under all future climate scenarios as shown in Table 5. That being said, the 16SrII-B and 16SrII-D subgroups phytoplasmas were registered in more than 25 plant hosts including economic crops, and medicinal and wild plants in Oman [1,3,9,18,40,41,42,43]. Therefore, studying the phytoplasma groups and the impacts of environment factors on these phytoplasmas in Oman ultimately will help the decision-maker in controlling the phytoplasma diseases and agricultural practices in Oman. In addition, the 16SrII-B and 16SrII-D distribution under various climatic projections could serve as a proxy of the host because the model is built on these environmental variables.

Furthermore, the results in Table 6 and Table 7 show that under different climatic scenarios, there were no similarities that could be attributed to factors such as the occurrence of data from samples collected from the northern part of the country. The selection of bio-environment variables might also be a source of uncertainty because there might be overlapping results. In addition, the global climate models used in this study were of coarse resolution, which could create uncertainties about their validity. However, this study has helped in analyzing the environmental variables that could contribute to the distribution of disease, thereby enabling the development of possible ways to stop, slow down, or reverse the negative impacts of climate change on 16SrII-D and 16SrII-B phytoplasmas. Therefore, studying the effects of climate change on 16SrII-D and 16SrII-B phytoplasmas is essential to establishing a reliable decision-making process to guide plant breeding research that can select the genetic strains best suited for specific areas in Oman. Moreover, this study will help decision makers determine suitable areas for growing acid lime trees and alfalfa in Oman during the coming 80 years.

MaxEnt proved its ability to make predictions about 16SrII-B and 16SrII-D distributions based on the environmental variables. This model can be used as a tool for land managers to predict the likelihood of presence of this disease based on small data samples.

## 5. Conclusions

This is the first study that evaluated the environmental variables that affect the 16SrII-D and 16SrII-B distribution of phytoplasmas diseases in Oman. In addition, this study has also predicted the effect of climate change on the distribution of the disease for the periods between 2021 and 2100.

1. The models produced reliable results based on the current distribution of the diseases. According to the model, isothermality (bio3), temperature annual range (bio7), minimum temperature of the coldest month (bio6), precipitation seasonality (bio15), and precipitation of the driest month (bio14) play a major role in the distribution of the WBDL (16SrII-B) phytoplasma disease. Similarly, isothermality (bio3) and precipitation of the driest month (bio14) played a significant role in AlfWB (16SrII-D) phytoplasma disease distribution. In addition, the mean diurnal range (bio2), seasonal precipitation (bio15), mean temperature of the wettest quarter (bio8), precipitation of the warmest quarter (bio18), and DEM played a significant role in 16SrII-D phytoplasma distribution.
2. On an overall basis, climate change will make more areas vulnerable to these two diseases. Therefore, this study will help in producing suitable strategies to control the disease spatial distribution and management.
3. The results generated in this research will be useful for Oman’s neighboring nations, where phytoplasma diseases are also prevalent.
4. This study identified hotspots and vulnerable areas that can help in mapping and delineating those places, and in developing new strategies to control the spread of the disease across Oman and other countries that face similar challenges.

Finally, this study would be the first attempt in spatial modeling of witches’ broom disease distribution in Oman. In other words, this study has opened windows of opportunities for research and development (R&D) in this area. Data collection and monitoring campaigns are important areas that efforts can be directed to, especially at local scales. Notably, in the nearest future, we will attempt further R&D in understanding the insect vectors of 16SrII-D and 16SrII-B phytoplasma disease. This will strengthen our understanding and providing a better picture of the distribution of phytoplasma disease in Oman under a changing climatic scenario.

## Figures and Tables

**Table 1:** The environmental variables considered in the 16SrII-D phytoplasma niche models and the average percent contributions of the environmental variables. The general statistics have been calculated with the help of occurrences (*n* = 68). The bold variables are the most frequently contributed factors of the model.

Variables	Percent Contribution	Permutation Importance	Min.	Max.	Mean	SD
Isothermality (Bio3)	29.7	1.1	36.8	50.4	43.82	2.79
Mean Diurnal Range (Bio2, °C)	25.6	18.1	8.8	14	11.77	1.26
Precipitation Seasonality (Bio15)	13.1	22.2	48.2	118	79.98	14.54
Mean Temperature of Wettest Quarter (Bio8, °C)	10.1	9.9	19.8	30.7	24.62	1.7
Precipitation of Warmest Quarter (Bio18, mm)	7	4.4	3	291	31	41.63
Precipitation of Driest Month (Bio14, mm)	5.6	32.3	0	11.1	2.23	1.91
Digital Elevation Data (m)	5.6	0.4	9	2062	418.92	356.5
Precipitation of Driest Quarter (Bio17, mm)	1.6	1.7	3	42.2	11.47	7.79
Annual Mean Temperature (Bio1, °C)	0.5	4.2	18.3	30	27.83	2.18
Annual Precipitation (Bio12, mm)	0.3	1.8	87.5	855	167.6	107.9
Precipitation of Wettest Quarter (Bio16, mm)	0.3	0.9	46.71	567	89.9	65.31
Precipitation of Coldest Quarter (Bio19, mm)	0.2	0.2	44.1	116	57.71	11.43
Mean Temperature of Coldest Quarter (Bio11, °C)	0.2	2.5	11.2	23.5	20.68	2.2
Max Temperature of Warmest Month (Bio5, °C)	0.2	0.2	31.5	45.1	40.87	2.61
Temperature Annual Range (Bio7, °C)	0.1	0	21.9	31.3	26.8	2.2
Mean Temperature of Driest Quarter (Bio9, °C)	-	0.1	15.2	37.5	29.8	4.57
Temperature Seasonality (Bio4)	-	-	411	661	544.4	59.51
Precipitation of Wettest Month (Bio13, mm)	-	-	18.2	263	38.13	30.76
Min Temperature of Coldest Month (Bio6, °C)	-	-	4.72	18.35	14.01	2.159
Mean Temperature of Warmest Quarter (Bio10, °C)	-	-	24.6	37.56	34	2.33

**Table 2:** The environmental variables considered in the 16SrII-B phytoplasma niche models and the average percent contributions of the environmental variables. The general statistics were calculated using occurrences (*n* = 63). The bold variables are the most contributed bioclimatic factors in the model.

Variables	Percent Contribution	Permutation Importance	Min.	Max.	Mean	SD
Isothermality (Bio3)	36.9	4.5	36.1	53.5	43.8	3.49
Temperature Annual Range (Bio7, °C)	24.1	16.1	15	30.3	25.7	2.50
Min Temperature of Coldest Month (Bio6, °C)	9.8	2.7	6.74	19.1	14.4	2.00
Precipitation Seasonality (Bio15)	7.9	16.5	43.7	107	74.9	15.2
Precipitation of Driest Month (Bio14, mm)	5.9	16.8	0	8.47	2.42	1.73
Mean Temperature of Driest Quarter (Bio9, °C)	3.2	24.9	33.2	43.9	40.14	2.129
Precipitation of Warmest Quarter (Bio18, mm)	3.1	0.1	2.14	172	34.2	31.5
Precipitation of Wettest Month (Bio13, mm)	2.3	0.8	15.8	143	36.2	18.5
Mean Temperature of Wettest Quarter (Bio8, °C)	2	3.9	18.2	32	24.6	2.70
Mean Temperature of Warmest Quarter (Bio10, °C)	1.7	5	26.4	36.8	33.6	1.9
Precipitation of Wettest Quarter (Bio16, mm)	1.2	0.3	41.4	31	86.3	40.4
Annual Precipitation (Bio12, mm)	0.6	0.1	88.4	535	171	82.1
Precipitation of Coldest Quarter (Bio19, mm)	0.5	0.4	12	124	58.7	17.0
Temperature Seasonality (Bio4)	0.4	4.9	211	655	522	79.5
Precipitation of Driest Quarter (Bio17, mm)	0.3	2.1	2	33.8	13.2	7.49
DEM (m)	0.3	0.8	9	1936	463	409
Annual Mean Temperature (Bio1, °C)	0	0	20.2	29.7	27.7	1.70
Mean Temperature of Coldest Quarter (Bio11, °C)	0	0	13.1	24.8	20.8	1.92
Max Temperature of Warmest Month (Bio5, °C)	0	0	17.2	35.3	28.7	3.56

**Table 3:** AUC values of II-B and II-D modeling distribution from various climate-change scenarios.

Period	AUC_mean_		AUC_mean_ Standard Deviation	
	16SrII-B	16SrII-D	16SrII-B	16SrII-D
2021–2040	0.8598	0.8268	0.0357	0.0469
2041–2060	0.900	0.837	0.058	0.044
2061–2080	0.931	0.858	0.026	0.038
2081–2100	0.913	0.894	0.043	0.024

**Table 4:** Percentage of distributional changes of 16SrII-B between current and future climate-change scenarios.

	Current 2021–2040	Current 2041–2060	Current 2061–2080	Current 2081–2100
Class	% Increase	% Increase	% Increase	% Increase
Gain	1.65%	2.21%	6.28%	24.74%
No Change	98.28%	97.67%	93.71%	75.22%
Loss	0.07%	0.12%	0.01%	0.03%

**Table 5:** Percentage of distributional changes of 16SrII-D between current and future climate-change scenarios.

	Current 2021–2040	Current 2041–2060	Current 2061–2080	Current 2081–2100
Class	% Increase	% Increase	% Increase	% Increase
Gain	0.08%	0.06%	0.05%	0.01%
No Change	88.36%	82.90%	79.66%	78.48%
Loss	11.56%	17.04%	20.30%	21.51%

**Table 6:** Predicted suitable areas for AlfWB disease (16SrII-D) under current and future climatic conditions.

Decade Scenarios	Predicted Area/km^2^				Percentage (%) of Increase/Decrease (Compared to the Current Distribution)			
	Total Unsuitable Habitat (0–0.40)	Total Poorly Suitable Habitat (0.40–0.60)	Total Moderately Suitable Habitat (0.60–0.75)	Total Highly Suitable Habitat (0.75–1.0)	Total Unsuitable Habitat	Total Poorly Suitable Habitat	Total Moderately Suitable Habitat	Total Highly Suitable Habitat
1970–2000 (current)	221,488.8	16,609.8	19,065	15,549.6	NA	NA	NA	NA
2021–2040	154,770.6	77,078.4	23,566.2	19,474.2	−24.91%	21.95%	1.58%	1.38%
2041–2060	227,812.8	30,504	8853.6	7719	1.66%	5.01%	−3.77%	−2.89%
2061–2080	247,845	20,050.8	4836	2157.6	8.94%	1.20%	−5.23%	−4.92%
2081–2100	264,380.4	7793.4	1581	1134.6	14.96%	−3.26%	−6.42%	−5.29%

**Table 7:** Predicted suitable areas for witches’ broom disease of acid lime trees (WBDL) disease (16SrII-B) under current and future climatic conditions.

Decade Scenarios	Predicted Area/km^2^				Percentage (%) of Increase/Decrease (Compared to the Current Distribution)			
	Total Unsuitable Habitat (0–0.40)	Total Poorly Suitable Habitat (0.40–0.60)	Total Moderately Suitable Habitat (0.60–0.75)	Total Highly Suitable Habitat (0.75–1.0)	Total Unsuitable Habitat	Total Poorly Suitable Habitat	Total Moderately Suitable Habitat	Total Highly Suitable Habitat
1970–2000 (current)	236,406	8481.6	17,614.2	10,211.4	NA	NA	NA	NA
2021–2040	235,457.4	10,750.8	19,846.2	8835	−1.03%	0.80%	0.76%	−0.53%
2041–2060	234,713.4	15,661.2	17,856	6658.8	−1.30%	2.59%	0.04%	−1.32%
2061–2080	227,422.2	18,897.6	20,125.2	8444.4	−3.95%	3.76%	0.86%	−0.67%
2081–2100	191,468.4	51,689.4	21,204	10,527.6	−17.03%	15.69%	1.25%	0.09%

## Data Availability

Data is contained within the article.
